# *Nosema ceranae* Can Infect Honey Bee Larvae and Reduces Subsequent Adult Longevity

**DOI:** 10.1371/journal.pone.0126330

**Published:** 2015-05-27

**Authors:** Daren M. Eiri, Guntima Suwannapong, Matthew Endler, James C. Nieh

**Affiliations:** 1 Division of Biological Sciences, Section of Ecology, Behavior, and Evolution, University of California San Diego, 9500 Gilman Drive, MC 0116, La Jolla, California, 92093–0166, United States of America; 2 Department of Biology, Faculty of Science, Burapha University, Chon Buri, 20131, Thailand; University of Camerino, ITALY

## Abstract

*Nosema ceranae* causes a widespread disease that reduces honey bee health but is only thought to infect adult honey bees, not larvae, a critical life stage. We reared honey bee (*Apis mellifera*) larvae *in vitro* and provide the first demonstration that *N*. *ceranae* can infect larvae and decrease subsequent adult longevity. We exposed three-day-old larvae to a single dose of 40,000 (40K), 10,000 (10K), zero (control), or 40K autoclaved (control) *N*. *ceranae* spores in larval food. Spores developed intracellularly in midgut cells at the pre-pupal stage (8 days after egg hatching) of 41% of bees exposed as larvae. We counted the number of *N*. *ceranae* spores in dissected bee midguts of pre-pupae and, in a separate group, upon adult death. Pre-pupae exposed to the 10K or 40K spore treatments as larvae had significantly elevated spore counts as compared to controls. Adults exposed as larvae had significantly elevated spore counts as compared to controls. Larval spore exposure decreased longevity: a 40K treatment decreased the age by which 75% of adult bees died by 28%. Unexpectedly, the low dose (10K) led to significantly greater infection (1.3 fold more spores and 1.5 fold more infected bees) than the high dose (40K) upon adult death. Differential immune activation may be involved if the higher dose triggered a stronger larval immune response that resulted in fewer adult spores but imposed a cost, reducing lifespan. The impact of *N*. *ceranae* on honey bee larval development and the larvae of naturally infected colonies therefore deserve further study.

## Introduction

Honey bees provide valuable pollination services for multiple agricultural crops [1,2]. However, despite the increasing global demand for this pollination service [3], problems with bee health have contributed to limiting the supply of colonies [4]. Each year since 2006, the USA has experienced consecutive overwintering colony losses of approximately 30% [5], and some European countries have reported similar losses [6,7]. Although the causes for these declines are not completely understood, researchers have identified multiple factors: mite infestation, pesticides, pathogens, and interactions between these factors [8,9].

We focus on a globally-distributed pathogen, *Nosema ceranae*, which significantly reduces the survival of bee colonies [10,11]. This microsporidian pathogen originally infected the Asian honey bee species, *Apis cerana* [12] and now also infects the European honey bee, *A*. *mellifera* [13,14]. The degree to which *N*. *ceranae* contributes to global colony losses is unclear: its effect on colony health varies between studies in different geographical areas [15,16], perhaps due to different environmental conditions [17]. However, multiple studies have demonstrated that *N*. *ceranae* infection decreases honey bee health [17], primarily by degenerating digestive tissue [18,19] and resulting in malnutrition and reducing lifespan [20,21]. In *A*. *cerana* and *A*. *florea*, infection reduces the protein content in hypopharyngeal glands [22]. Flight behavior can also be impaired by infection, which may reduce forager numbers and colony food intake [23]. In addition, synergistic interactions between pesticide exposure and *N*. *ceranae* infection can increase susceptibility to *N*. *ceranae* infection [9,24] and mortality [25,26].

*Nosema ceranae* is believed to infect only adult honey bees, although *Nosema* species can infect larvae of other insect species, including a close relative of honey bees, bumble bees [27] (tested by inoculating larvae with spores [28]). Larval Lepidoptera [29] and Coleoptera [30] can also be infected by different *Nosema* species. Surprisingly, no published studies, to date, have directly tested if *N*. *ceranae* can infect honey bee larvae, although there is some evidence for larval infection. Traver & Fell detected low levels of *N*. *ceranae* DNA in honey bee queen [31] and drone [32] larvae.

Transmission of *N*. *ceranae* is poorly understood. Spores are exclusively produced in midgut tissues [33]. However, spores have been detected in corbicular pollen [34]. Recently, Traver and Fell [31] detected *N*. *ceranae* DNA in royal jelly from hives naturally infected with *N*. *ceranae*. Thus, larval food could provide a natural infection route. However, even if *N*. *ceranae* is not directly transmitted through brood food, nurse bees feed larvae orally [35] and oral transmission can occur between adults [36]. Such oral transmission may arise from fecal spores traveling to the mouthparts of the food recipient, but it nonetheless demonstrates that a bee obtaining food from an infected bee can also be infected by *N*. *ceranae*.

The evidence that *N*. *ceranae* cannot infect *A*. *mellifera* larvae is largely indirect. Newly emerged adults from *Nosema*-infected colonies are reportedly uninfected, as measured through gut spore counts [37]. However, in these newly emerged bees, *Nosema* may still be in the intracellular life stage (actively reproducing vegetative state) that later produces the mature spores seen in older bees. Nurse bees may also behave hygienically, removing brood that is heavily infected, as they do in colonies infected with *Paenibacillus larvae* [38] or with the fungal pathogen, *Ascophaera apis* [39]. Finally, sick bees that emerge may remove themselves from the colony. Rueppell et al. [40] demonstrated this phenomenon in young adult bees sickened with drug or CO_2_ treatments. Goblirsch et al. [41] found that workers infected with *N*. *ceranae* were twice as likely to engage in early foraging, causing them to spend more time outside of the colony. It is also possible that honey bee larvae are relatively more resistant to *N*. *ceranae* infection than adults. However, given the evidence for larval infection in the closely related bumble bees and other taxa, it is reasonable to ask if *N*. *ceranae* can infect honey bee larvae.

We therefore tested the larval infection hypothesis by directly infecting *A*. *mellifera* larvae with *N*. *ceranae*. We used a single dose of *N*. *ceranae* spores given only once to larvae in their brood food. To exclude the possibility of hygienic bees removing infected larvae, we used *in vitro* rearing. We hypothesized that the midgut cells of pre-pupae infected as larvae would show proliferating spores and therefore used histology [42] to check for intracellular spore development. A separate set of treated bees were reared to adulthood and maintained in cages to measure their longevity and to eliminate the possibility of infected adult self-removal. We hypothesized that larvae receiving the *N*. *ceranae* treatment would become infected, that pre-pupae and adult stages would contain *N*. *ceranae* spores in their midguts, and that this infection would decrease adult longevity. Finally, larvae were fed autoclaved spores as a control to test the possibility that spores counted in pre-pupae and adults were residual spores from the treatment, not the result of infection.

## Results

### Histology

**Fig 1:**
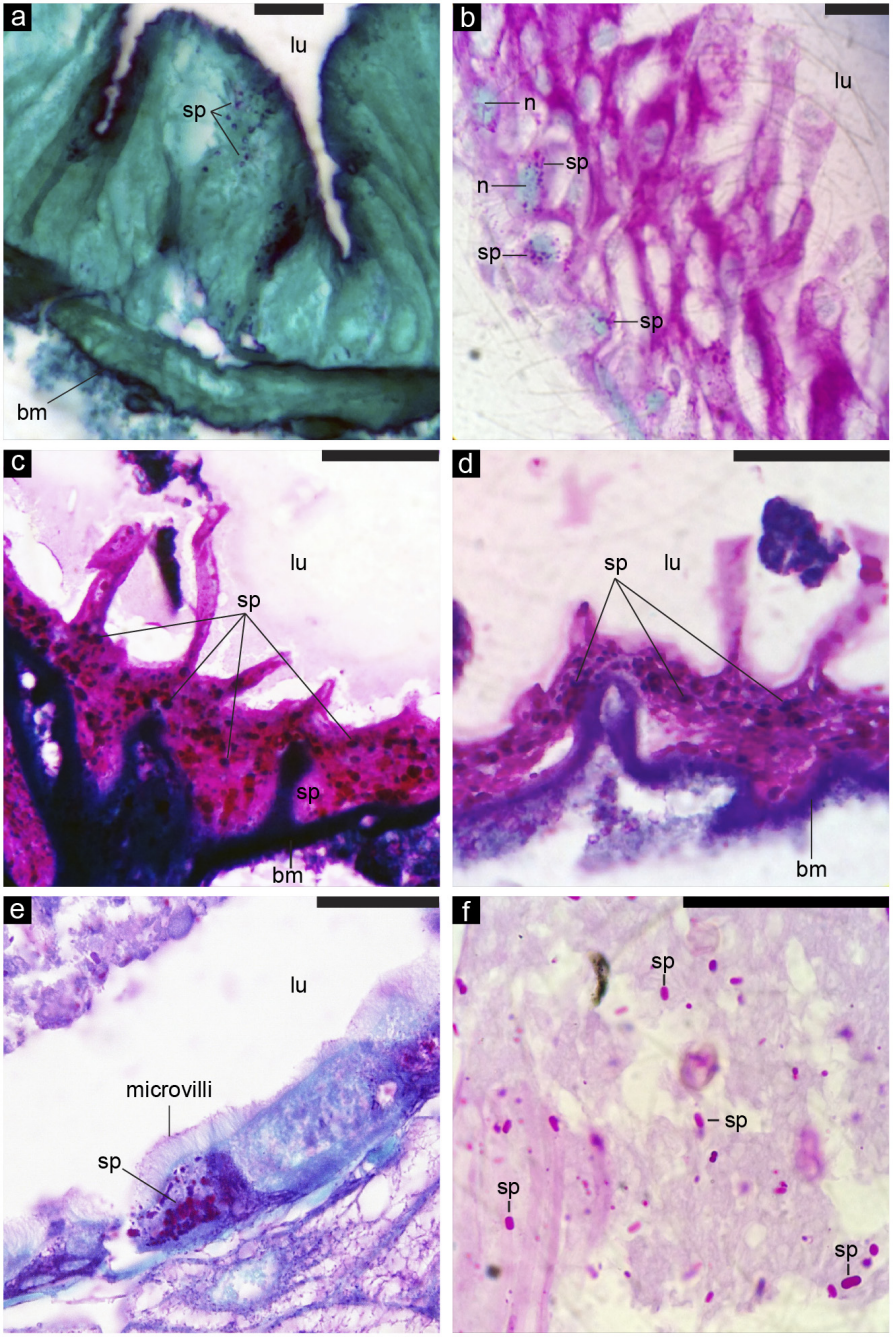
*Nosema* spores developing intracellularly in the midgut cells of bees at an early pre-pupal stage. (a-f) Images are shown at relative magnifications from 400-1400X, and scale bars correspond to 50 μm. The abbreviations refer to the following: lu = lumen (interior of midgut), bm = basement membrane, sp = *Nosema* spore, and n = midgut cell nucleus (relatively large blue-green ovoid structures shown in part b).

At the pre-pupal stage (8 days after egg hatching), 41% of bees fed as larvae (3 days after egg hatching) showed spores developing intracellularly in bee midgut cells (*n* = 35 bees from two colonies). Fig 1a–1e shows densely packed spores that have propagated inside midgut cells. Fig 1b shows a classic clustering of developing spores around the midgut cell nucleus. In infected bees (individuals with at least one intracellular *N*. *ceranae* spore in a midgut cell), 52±2% of these midgut cells were infected (range of 20–100% of cells infected). *Nosema ceranae* can therefore infect honey bee larval midgut tissue.

### Pre-pupae infection levels

We reared 220 first instars (1 day after egg hatching) from three colonies to the pre-pupal stage. We gave larvae *N*. *ceranae* spores at 3 days after egg hatching. Overall, 55% and 60% of larvae treated with a single 10K or 40K spore dosage, respectively, had midgut spores as pre-pupae (Table 1). The maximum number of spores per bee was 12,500 and 15,000 for 10K and 40K treatments, respectively, and only 1.4% of control larvae fed no spores had spores present as pre-pupae (maximum of 2,500 spores per bee). None of the bees fed autoclaved spores had any spores in their midguts as pre-pupae.

**Table 1:** Detailed spore count data for pre-pupae and adults. Mean spore counts ± 1 standard error are shown in Fig 2a and 2b.

Stage	Treatment	# of individuals	Percentage with spores present (%)	Range of spore counts
Pre-pupae	Control _fed no spores_	70	1.4	0–2500
	Control _fed autoclaved spores_	20	0	0
	10K	67	55.2	0–12500
	40K	63	60.3	0–15000
Adult	Control _fed no spores_	129	3.1	0–10000
	Control _fed autoclaved spores_	55	0	0
	10K	93	66.7	0–215000
	40K	109	44.0	0–295000

**Fig 2:**
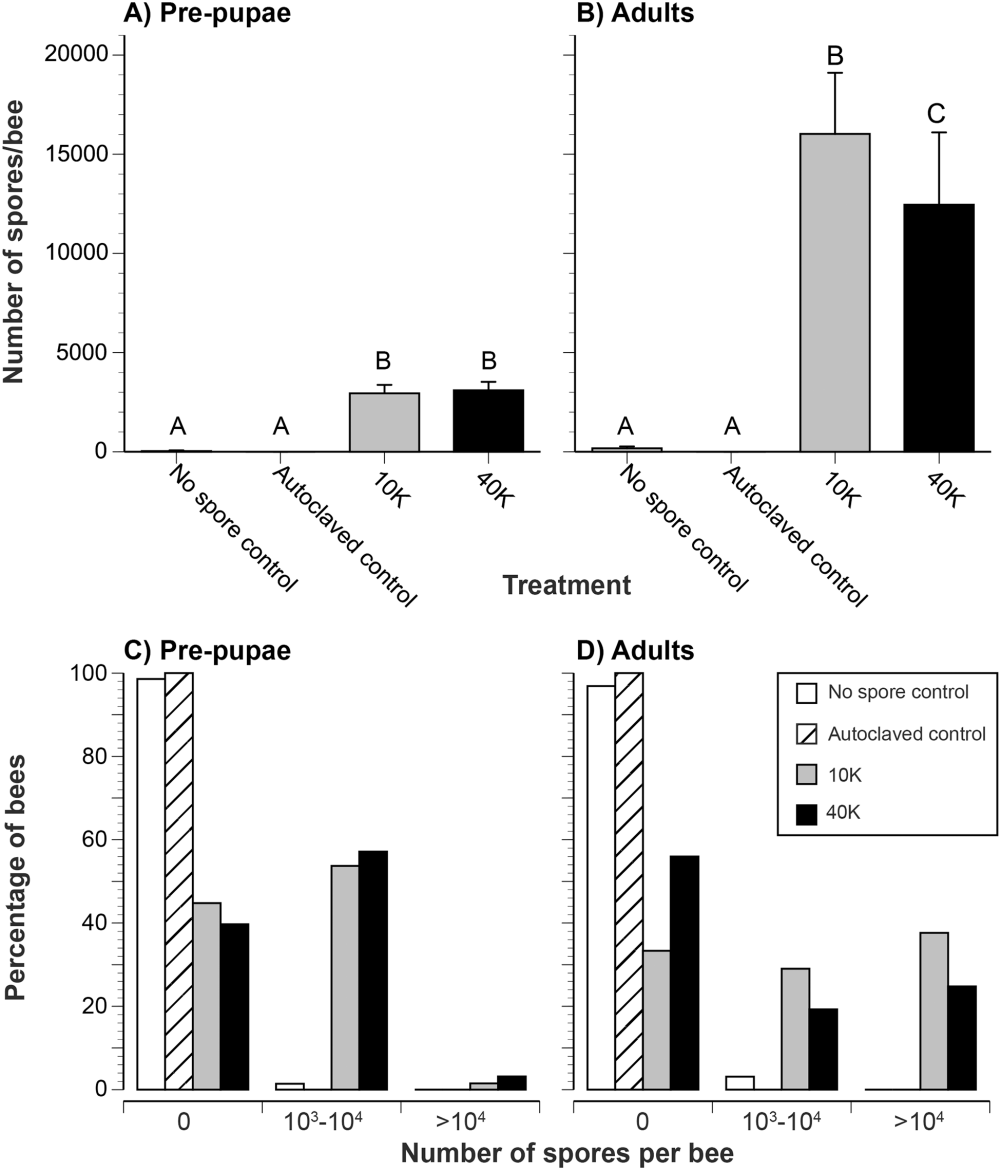
*Nosema ceranae* spore counts in pre-pupae and adults. Effect of larval exposure to *N*. *ceranae* on midgut spore count in (a) pre-pupae and (b) adults upon death. (a) The average number of spores per bee midgut is shown. Error bars show standard errors. Different letters indicate significantly different treatments. (b) A higher percentage of bees fed *N*. *ceranae* as larvae were infected as (c) pre-pupae and (d) adults. These graphs show the percentage of bees with different levels of infection.

The 10K and 40K larval treatments resulted in significantly elevated pre-pupal spore counts as compared to control bees fed no spores or autoclaved spores (Table 1, Fig 2a; Kruskal-Wallis: *χ* ^*2*^ = 72.29, df = 3, *P* < 0.0001; Steel-Dwass: Z > 4.16, *P* ≤ 0.0002). There is no significant difference in pre-pupal spore counts between 10K and 40K treatment groups (Steel-Dwass: Z > 0.39, *P* = 0.92). There is no significant difference in spore counts between larvae fed no-spores or autoclaved spores (Steel-Dwass: Z = -0.51, *P* = 0.96). In addition, respectively 1.4% and 0% of larvae fed no spores or autoclaved spores had spores as pre-pupae. In contrast, 55% and 60% of larvae respectively fed 10K and 40K spores were infected as pre-pupae (Table 1, Fig 2c).

### Survival to adult emergence

To determine if *N*. *ceranae* treatment at the larval stage affects survival to adult emergence, we reared 551 individuals to adulthood from five colonies. Among the three treatments (control, 10K, and 40K), there is no significant difference in the number of larvae that survived to emerge as adults: 89%, 78%, and 72% of larvae in the control, 10K, and 40K treatment groups survived to adulthood (*G*_*pooled*_ = 3.00, *P* = 0.22, df = 2, *n* = 551 bees). There are no significant differences between trials (*G*_*heterogeneity*_ = 11.64, df = 10, *P* = 0.31, *n* = 6 trials).

### Adult emergence mass

We found no significant effect of treatment upon adult emergence mass (*F*_2,404_ = 0.47, *P* = 0.63). The average masses of adults upon emergence are respectively 135.0 ± 2.0, 134.2 ± 2.1, and 132.1 ± 2.3 mg for the control, 10K, and 40K treatments.

### Adult infection levels at death

We counted the number of spores per adult at death in 386 individuals from seven colonies (Table 1). There is a significant effect of treatment: larvae that were fed a single dose of *N*. *ceranae* spores subsequently had significantly elevated spore counts as adults (Kruskal-Wallis: *χ* ^*2*^ = 137.01, df = 3, *P* < 0.0001; Fig 2b). A few individuals treated with *N*. *ceranae* spores contained up to 295,000 spores upon adult death. Surprisingly, 10K treated larvae had 1.3 fold more spores upon adult death than 40K treated larvae. This difference was statistically significant (Steel-Dwass: *Z* = -3.01, *P* = 0.0073; Fig 2b). Similarly, a higher percentage of adult bees (67%) were infected (had non-zero spore counts) from the 10K larval treatment as compared to the 40K larval treatment (44% of adult bees). Only 3% of control bees had spores (maximum of 10,000 spores per bee). None of the larvae fed autoclaved spores had any midgut spores as adults. Both 10K and 40K treatments resulted in significantly elevated spore counts compared to both control treatments: Steel-Dwass: *Z* > 5.72, *P* ≤ 0.001).

### Adult longevity

**Fig 3:**
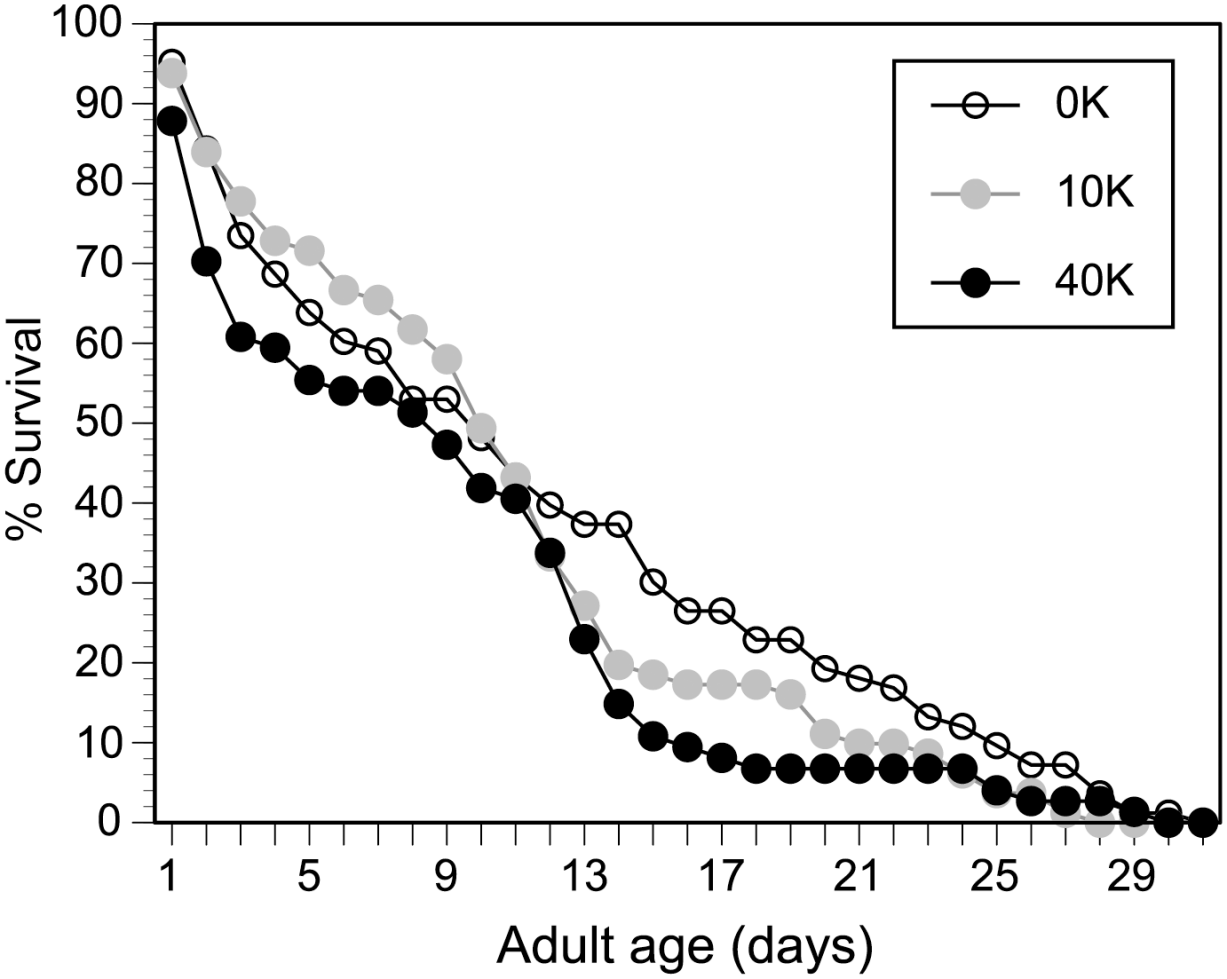
Effect of larval exposure to *N*. *ceranae* on adult survival. Survival curves for the treatments are shown. A total of 238 individuals from four different colonies emerged as adults and were monitored in cages to measure survival.

We monitored 238 adult bees, reared *in vitro*, until they died in incubated cages, providing water, sterilized sucrose (2.0M), and pollen mixed with sucrose *ad libitum*. Larval exposure to a single 40K spore dose significantly reduced adult longevity as compared to the control (Fig 3; Survival analysis, Log-Rank test, *χ* ^*2*^ = 4.64, df = 1, *P* = 0.03). The age at which 75% of adults died is respectively 18 days, 14 days, and 13 days for the control, 10K, and 40K treatments. Thus, a 40K treatment decreased the age by which 75% of adult bees died by 28% as compared to controls. There is no significant difference in adult lifespan between the control and 10K or between the 10K and 40K treatments (Fig 3; Survival analysis, Log-Rank test, *χ* ^*2*^ < 1.79, df = 1, *P* ≥ 0.18).

## Discussion

*Nosema ceranae* infection contributes to poor honey bee health globally and is thought to only infect adult honey bees. However, by using controlled *in vitro* exposure to spores in brood food, we show that *N*. *ceranae* can infect *A*. *mellifera* larvae. A single exposure to 10K or 40K *N*. *ceranae* spores during larval development resulted in low levels of pre-pupal infection and elevated adult infection (Fig 2). By the early pre-pupal stage, spores visibly developed intracellularly in bee midgut cells (Fig 1). *Nosema* infects by injecting sporoplasm, not whole spores, inside midgut cells [33]. Thus the presence of spores inside midgut cells is a result of an active, propagating infection. In addition, the presence of fully formed spores packed inside midgut cells (Fig 1) demonstrates that *N*. *ceranae* can develop through its full life cycle in larvae and is not halted at merogony or early sporogony.

We fed larvae with a 40K spore dose and then used histology to examine their midgut cells 5 days later. In infected bees (41% of bees had at least one intracellular *N*. *ceranae* spore in a midgut cell), 52±2% of these midgut cells were infected. In comparison, Higes et al. [43] fed newly emerged *A*. *mellifera* workers with *N*. *cerana*e (125,000 spores/bee) and showed that 66% and 82% of their midgut cells were respectively infected 6 and 7 days after infection. Suwannapong et al. [44] fed newly emerged *A*. *cerana* workers with a 40K dose of *N*. *ceranae* spores and reported that 34% of bee midgut cells were infected 6 days later (infection percentage converted from the reported infection ratio). The percentage of infected midgut cells increases with spore dose [44], which is 3-fold higher in Higes et al. [43] as compared to Suwannapong et al. [44]. Thus, the percentage of infected pre-pupal midgut cells in our study, given our 40K dose, is lower than the values reported by Higes et al. [43] in newly emerged *A*. *mellifera* adults. These differences may arise from differences in initial spore doses, the bee species and *Nosema* species used, bee immune responses at different developmental stages, other physiological factors, or a combination of these factors [17,45].

We used a standard method, midgut spore counts, to assay infection levels [17]. Real-time PCR provides another method of detecting spores and different life stages of *Nosema*. However, *Nosema* is spread through spores [17]. Thus, counting the number of spores in the midgut provides a direct measure of how infectious a bee will be. It is possible that some of the spores counted in pre-pupae and adults were original, un-germinated spores given to larvae and were not the result of sporogenesis in the larvae. However, this is unlikely to explain our results. First, the spores (10 μl in volume) were only provided in brood food (100 μl) given to larvae 3 days after egg hatching. Each day, we aspirated out the old food and provided new food that did not contain spores. Each larva therefore had four changes of food, substantially diluting any residual spores in its growth chamber and limiting surface contamination. When the larvae began to produce uric acid crystals, a sign of initial defecation [46], we gently blotted them dry with sterile tissues and moved them to sterile pupation plates where we allowed them to completely defecate over the next 24 hours. We then carefully dissected out the midgut only and counted the spores on a hemocytometer [47]. Most importantly, control larvae fed 40K autoclaved spores and treated in exactly the same way as larvae given live spores all showed 0 spore counts as pre-pupae and upon adult death (Table 1). Thus, the pre-pupae spore counts were the result of infection by living spores.

Throughout our experiment, accidental contamination through daily handling or through the bees’ artificial diet was evidently minimal. Only one of the 70 control pre-pupae had spores, estimated at 2,500 spores. Very few of our adult bees in the control group were found to have spores present in their midgut (Table 1) and adult infection levels were significantly higher in the 10K and 40K treatment groups than in the control group (Fig 2b).

Because adults were held in group-cages, infected adults could have passed on spores to other adults in the same cage. However, each cage only contained adults from the same treatment of the same trial (maximum of 12 bees per cage). Any spores shared between adults would therefore have arisen from an infection started by the original one-time larval dose. Cross-cage contamination was not a factor because control bees were essentially uninfected as adults (Table 1). Such adult spore exchange could have homogenized infection levels among adult bees within a cage, but the average infection level should still reflect the degree of adult infection caused by the larval treatment.

Finally, *N*. *ceranae* increases food consumption in adult bees [20,50], we therefore expected that larvae infected by *N*. *ceranae* would be heavier at adult emergence. However, we did not find any significant effect of treatment on body mass, which was 133.9 ± 2.0 mg (for all treatment groups) similar to the 138.9 ± 2.0 mg of emerging adults reported by another *in vitro* study [51].

### *Nosema* species infect larvae of other insects

The previously reported inability of *Nosema* to infect honey bee larvae is surprising because *Nosema* can infect larvae of other insects. In the Lepidoptera, *N*. *algerae*, *N*. *ploidiae*, *N*. *pyrausta*, and *N*. *bombycis* respectively infect the larvae of *Heliothis zia*, [29]; of the Indian-meal moth, *Plodia interpunctella* [52]; of the European corn borer, *Ostrinia nubilalis* [53]; and of the silk moth, *Bombyx mori* [54]. In the Coleoptera, *N*. *whitei* infects the larvae of flour beetles, *Tribolium castaneum* [30] and *Tenebrio molitor* [55]. Most relevantly, in the Hymenoptera, *N*. *bombi* can infect bumble bee (*Bombus terrestris*) larvae [28]. A separate study confirmed this, showing that *B*. *terrestris* queens inoculated as larvae with 313,000 *N*. *bombi* spores developed nosemosis and were less able to found colonies [56]. A field study revealed additional fitness consequences. Infected *B*. *terrestris* queens produced fewer workers, drones, and virgin queens than controls [57]. In addition, *N*. *bombi* DNA is found in eggs of the bumble bee, *B*. *lucorum*, suggesting vertical transmission [58]. Similarly, Traver & Fell [31] detected low levels of *N*. *ceranae* DNA in honey bee queen larvae. Our finding that *N*. *ceranae* can infect *A*. *mellifera* larvae (Figs 1 and 2) therefore fits with the known ability of *Nosema* spp. to infect larvae in a wide variety of insects, including the Apidae.

### Evidence for and against larval infection of *Apis mellifera*

Traver & Fell [31] provided evidence that *N*. *ceranae* can infect honey bee larvae by detecting low levels of *N*. *ceranae* DNA in larvae and newly emerged queens. However, Bailey [59] and Smart & Sheppard [37] found no spores in recently emerged bees originating from infected colonies. This may not be surprising because Meana et al. [60] reported lower and highly variable spore counts in younger bees as compared to older bees in infected colonies. Hassanein [61] added spores of a different species of *Nosema*, *N*. *apis*, to combs containing larval honey bees and then placed these brood combs back into a nest infected with *N*. *apis*. None of the resulting pupae or adult bees were infected. Thus, *N*. *apis* may not infect *A*. *mellifera* larvae, although removal of infected larvae by hygienic bees could also yield this result.

*In vitro* rearing allowed us to control for potential hygienic behavior and may have reduced larval health, increasing their susceptibility to *N*. *ceranae* infection. However, 89% of our control larvae survived to adulthood. In natural rearing by full colonies, 85% of eggs laid by the queen survive to become to adult workers [62]. Thus, our artificial rearing conditions resulted in an excellent survival rate when compared to natural rearing. It is possible that some aspect of our bee diet may have increased bee susceptibility to *N*. *ceranae* infection [63]. For example, raising larvae on an artificial diet may have reduced the diversity of pollen protein sources that they received. However, the protein in the larval diet came from natural royal jelly and, upon adulthood, bees were fed natural pollen harvested from diverse sources by honey bees. Even if our diet facilitated *N*. *ceranae* infection, our results still demonstrate that this pathogen can infect honey bee larvae. Honey bees are increasingly exposed to a wide variety of stressors from diet, diseases, parasites, and chemicals [64–66], and thus the ability of *N*. *ceranae* to infect even potentially stressed larvae remains relevant.

### Adult infection levels

Multiple conditions can affect spore infectivity [17], and studies report a wide range in infectivity [13,17,37,67]. Although spore counts obtained from randomly sampled bees in a colony may not be a good measure of colony health, measuring spore counts in older bees (foragers) is recommended as a more reliable measure of colony infection levels and health [60]. We therefore reared our bees to their maximum adult lifespan (Fig 3) and only measured spore counts upon adult death.

In terms of the percentage of infected bees, Forsgren & Fries [68] gave young adult *A*. *mellifera* workers a 10K *N*. *ceranae* dose and found that they were all infected after 12 days. Suwannapong et al. [22] treated *A*. *florea* adults and reported that 45% and 68% were infected after 14 days when treated with 10K and 40K spores, respectively. In our experiment with *A*. *mellifera*, the percentage of infected adults was 66% and 44% for 10K and 40K larval treatments, respectively (Table 1).

With respect to spore counts per bee, bees from both *N*. *ceranae* treatments were significantly infected compared to controls (Fig 2). In naturally infected colonies, the mean spore count was 13,400 spores/bee for house bees (≤21 days old) and 2,380,000 spores for foragers (≥22 days old) [37]. In the US, average spore counts in naturally infected colonies range from 564,000 to 800,000 spores per bee [8]. In our study, although some individuals were infected with more than 200,000 spores, average infection levels were low (16,021 and 12,454 spores per bee for the 10K and 40K treatments, respectively) when compared to experimental studies in which adult bees, not larvae, were infected. However, most of these studies exposed adults at much higher doses. When adult *A*. *mellifera* were fed approximately 100,000 spores/bee (2.5 fold more spores than our highest treatment), the average infection level after six days was 570,000 spores/bee [13]. Finally, major gut reorganization during honey bee metamorphosis [48,49] could affect subsequent levels of adult infection, particularly if such reorganization reduces infection levels. This remains to be determined.

### Longevity effect

Our longevity results also show a smaller decrease in longevity for bees infected as larvae (Fig 2) than for bees infected as adults [41]. Goblirsch et al. [41] infected newly emerged adults with 10K *N*. *ceranae* spores and showed a 21% decrease in the age at which 75% of bees died and a 36% decrease in the median age of death. In our experiment, a 40K treatment decreased the age by which 75% of adult bees died by 28% and the median age of death by 10% (Fig 2). However, even relatively small changes in longevity may have a cumulative effect, particularly if the colony’s health is challenged by other factors [24].

Both 10K and 40K treatments resulted in significant adult infection compared to controls, but larvae given the lower (10K) dose had 1.3 fold more midgut spores upon adult death than larvae given the higher (40K) dose. We speculate that the unexpectedly stronger effect of the lower spore dose may have arisen from larval immune responses. A 10K spore dose per bee is an order of magnitude lower than the dose typically fed to infect adult bees [13,69] and may not have fully activated an immune response, allowing individuals to be infected. The 40K dose may be large enough to trigger a stronger immune response, resulting in equal infection levels at the pupal stage and culminating in a lower spore count (than the 10K treatment) upon adult death. Alternatively, infection levels should increase as bees age [37]. It is thus possible that the 40K dose resulted in lower spore counts because these bees had a slightly younger median age of death (13 days) compared to 10K-treated individuals (14 days). However, there is no significant difference between the longevity of bees receiving the 10K or 40K treatments (see Results). A future study is required to test this immune response hypothesis.

### Summary

Our data suggest the following pattern of infection: relatively low spore production in larvae and pre-pupae followed by a higher rate of spore proliferation in adult bees. However, this should be verified in the field with colonies naturally infected with *N*. *ceranae*. Although our results demonstrate that larvae can be infected, future studies with field colonies are necessary to determine what spore doses larvae are exposed to and if larval infection is common or rare. Finally, it is interesting that *N*. *ceranae* is highly prevalent and somewhat resistant to treatment in warmer climates [17] where brood production, to varying degrees, can occur year-round. Could brood infection by *N*. *ceranae* result provide a residual, time-delayed source of hive infection?

## Materials and Methods

### Ethics Statement

Bee colonies located at the University of California, San Diego Biological Field station required no permits. Appropriate permission was obtained from the UC Natural Reserve System for bee colonies located at the Elliott Chaparral Reserve.

### Spore preparation

*Nosema ceranae* spores were originally obtained from infected *A*. *florea* and *A*. *cerana* workers in Chon Buri, Thailand and fed to *A*. *mellifera* workers in La Jolla, California to ensure a fresh stock, renewed weekly, for our experiments. Spore-producing bees were not fed pollen, only pure 2.0 M sucrose solution (55% sucrose w/w) to ensure that gut contents consisted mainly of spores. To obtain spores, we dissected out adult honey bee midguts, homogenized them in sterile double distilled water (ddH_2_0), and vacuum-filtered them through Fisherbrand P8 filter paper with 20–25 μm pores. We collected the filtrate in microcentrifuge tubes that we centrifuged (Eppendorf 5415D centrifuge) at 9279 g (Relative Centrifugal Force) for 15 min, a modified method of Webster et al. [70]. We then removed the supernatant and resuspended the pellet in sterile ddH_2_0 water. This procedure resulted in fairly pure spore preparations as determined with a microscope. We measured spore concentrations with a hemocytometer in a compound microscope (Zeiss Axioskop), making two independent measures of each sample and recording the average spore count [47]. We made appropriate dilutions with sterile ddH_2_O to obtain the necessary treatment doses.

DNA was extracted to confirm we were using *N*. *ceranae*. We centrifuged dissected honey bee gut tissues with an estimated 40,000 spores per microliter at 9279 g to form a pellet. We then crushed the pellet using liquid nitrogen and extracted DNA with the Bioneer Accuprep Genomic DNA extraction kit. Using standard PCR methods, we sequenced the resulting spore DNA, using Genbank sequences to confirm their identity. DNA extracts for *N*. *ceranae* were screened using primer pairs NoscRNAPol-F2 and NoscRNAPol-R2 [71]. All PCR reactions were carried out in 20 μl reactions containing at least 5 ng total DNA, 10X PCR buffer (750 mM Tris-HCL [pH 8.5], (NH_4_)_2_SO_4_ (Apex Life Science company), 0.25 mM of each dNTP (Promega), 0.5 μM of each primer (Allele Biotech), 1% Tween, 50 mM MgCl_2_, and 1 unit of Taq DNA polymerase (Apex Life Science Co.). Cycling conditions were an initial 5 min at 95°C, then 35 cycles of 1 min at 95°C, 1 min annealing at 58°C, 1 minute at 72°C, and a final 10 min extension at 72°C. The 662 base pair amplicon was separated through gel electrophoresis and visualized using SYBR-safe (New England Biolabs). DNA was sequenced by Retrogen, Inc. with an Applied Biosystems 3730xl DNA Analyzer.

### Infecting honey bee larvae

The standard developmental timeline for a honey bee is as follows: 1, 7, and 19 days after egg hatching, the bee respectively becomes a larva, a pre-pupae, and finally an adult worker [72]. Our experiment used the following timeline: we grafted first instars (1 day after egg hatching), and inoculated individuals with the treatment or control when they were 3 days old after egg hatching. Once larvae had become pre-pupae, we dissected out their midguts at 8 days after egg hatching.

Methods for rearing larvae *in vitro* were modified from Huang [46]. Honey bee larvae for the different experiments were obtained from eight different colonies located at the University of California, San Diego Biological Field Station and the Elliott Chaparral Reserve, between November 2011 and March 2013. Larvae (1 day after egg hatching) were grafted individually into 24-well cell culture plates containing 100 μl of basic larval diet (BLD, see below) in each cell with a sterilized bamboo grafting tool. We maintained sterile conditions, carrying out all grafting, feeding, and transferring in a sterile laminar flow hood (AirClean 600). All equipment, including incubators, cages for holding adults, cell culture plates, glassware, and pipettes were regularly sterilized with bleach (10% solution, soaking for at least 30 min followed by repeated rinses with deionized water), followed by 70% ethanol, dried in the sterile hood, and sterilized with ultraviolet light for 1 hr. Researchers wore gloves sterilized with 70% ethanol. All pipette tips and tissues used were autoclaved. We collected and autoclaved all waste to reduce *N*. *ceranae* contamination of the environment.

All larvae were fed a basic larval diet (BLD) consisting of 50% royal jelly (Stakich, Inc., Royal Oak, Michigan, USA 48073), 37% distilled water, 6% D-glucose, 6% D-fructose, and 1% yeast [46,73]. Royal jelly was not analyzed for the presence of *N*. *ceranae*, but all treatments were fed the same diet and individuals treated with the control had minimal spores detected in pre-pupae and in adults. We boiled distilled water, cooled it to 34°C, and then added glucose, fructose, and yeast. Defrosted royal jelly and the remaining ingredients were then combined and thoroughly mixed to the correct concentrations. This BLD was stored at 4°C and used for up to three days. Before feeding, BLD was warmed to 34°C.

It is unclear what spore dose larvae may naturally be exposed to. However, our goal was to test if larvae could be infected by *N*. *ceranae*. We therefore infected larvae with three different spore doses, a low dose of 10,000 (10K) spores, a higher dose of 40,000 (40K) spores, and an additional 40,000 spore dose that was autoclaved (40K autoclaved). Although previous studies have inoculated adult bees with larger doses [45,74], 10,000 spores is a standard minimum dose [75] that has been shown to produce infection [41,44,68]. This low dose also reduces the possibility of counting non-germinated spores as spores produced by infection [75]. The autoclaved spore treatment is also designed to test this potential issue.

Larvae were kept in a dark incubator maintained at 34°C and 90% humidity when not being fed. The BLD was removed by aspiration and the following amounts of new BLD were added daily to each cell: 100 μl (for larvae ≤ 3 days old after egg hatching) and 200 μl (up to pre-pupal stage). When larvae were 3 days old (after egg hatching), we added 10 μl of each of the following treatments prepared in sterile distilled water to each larva’s share of BLD: control (0K) spores/bee, 10,000 (10K) spores/bee, 40,000 (40K) spores/bee, or 40,000 (40K autoclaved) autoclaved spores/bee. Because it is not possible for experimenters to orally feed larvae, each larva could have consumed fewer spores. These doses therefore represent the maximum number of spores that a larva could have consumed. However, all larvae were given 10 μl of distilled water containing the same number of spores (within each treatment dose) in 100 μl of fresh BLD after old BLD had been removed by aspiration.

Once larvae entered the pre-pupal stage (identified by the uric acid crystal excreted by bees 7 days from egg hatching) we prepared new, sterile 24-well cell culture plates, with each cell containing an autoclaved dust-free tissue (Kimwipes, 3 cm x 3 cm strips). Pre-pupae were delicately cleaned with sterile tissues and transferred to new cell culture plates, which were kept in a dark environment maintained at 34°C and 75% humidity.

### Histology

Larvae from two colonies were obtained, grafted, and treated with the 40K spore dose at three days old, after-egg hatching. Once individuals defecated and became pre-pupae (day 8 from egg hatching), the midguts were dissected out and the tissue was fixed using Bouin’s fluid solution for 24 hours, followed by three washes with 70% ethanol, dehydrated with a standard 70%-100% ethanol series, and embedded in melted paraffin. Using a rotary microtome (Leica, Germany), we sliced the midguts into 6 μm thick cross sections, stained with Periodic acid-Schiff (PAS) stain, and used Light Green Counterstain [44]). All sections were then examined using a compound light microscope. For each bee, we randomly selected three cross-sections and counted the number of infected and uninfected midgut cells in each section. An infected cell has at least one intracellular *N*. *ceranae* spore. We then calculated the average percentage of infected midgut cells per bee.

### Adult honey bees

Once the honey bees reared *in vitro* emerged as adults, we weighed them (Mettler AE200 scale), placed them into sterilized ventilated clear plastic cages (12 cm x 8 cm x 12 cm), separated by treatment (12 bees per cage), and fed sterile 2.0 M sucrose, sterile water, and grounded up pollen mixed with sucrose (30% 2.0M sterile sucrose and 70% pollen w/w) *ad libitum* [76]. All solutions were sterilized by autoclaving. The pollen (Betterbee Inc., Greenwich, New York, 12834) was obtained from honey bee corbiculae and was irradiated to kill potential pathogens. Adults were allowed to live as long as possible. Each day, we removed dead bees, recorded mortality, and changed the pollen-sucrose food mixture. Dead bees were kept at -18°C until their midguts were removed. Cages were maintained in a dark environment at 34°C and 70% humidity. After all bees in a cage had died, we removed the cage and sterilized it as described above.

### Counting spores

We allowed pre-pupae to defecate (day 7 from egg hatching), cleaned them gently with sterile Kimwipes and placed them into new clean and sterile cell culture plates where they pupated for another 24 hours [77]. Pre-pupae were then frozen at -80°C. After thawing, we used dissecting tools that we meticulously cleaned with bleach, ethanol, and rinsed multiple times with water between each bee. We carefully cut open each bee (pre-pupa or adult upon death, depending upon the experiment) and gently pulled out the midgut through the base of the abdomen [75], placing the contents in 100 μl of distilled water in a centrifuge tube. We homogenized the gut contents and then pipetted out 7μl into a hemocytometer to count spores [47]. Two different samples were counted and then averaged to determine the number of spores per individual.

### Statistical analysis

We used G-tests (calculated with Microsoft Excel v14.3.9) to test if the number of larvae surviving to emerge as adults varied among treatments. After confirming that our adult emergence mass data met parametric assumptions, we used ANOVA to analyze our mass data. We used a Kruskal-Wallis test and Steel-Dwass post-hoc comparisons test to determine the effect of our treatments on *N*. *ceranae* spore counts in pre-pupae and adults. We tested the effect of treatment on adult longevity with non-parametric Kaplan-Meier survival analyses (Log-Rank tests). All other tests were performed using JMP 10.0 statistical software. We report averages as mean ± 1 standard error.

## References

[1] Morse RA, Calderone NW. The value of honey bees as pollinators of US crops in 2000. Bee Culture. 2000;128: 1–15.

[2] Winfree R, Gross BJ, Kremen C. Valuing pollination services to agriculture. Ecol Econ. 2011;71: 80–88. 10.1016/j.ecolecon.2011.08.001 21429164

[3] Aizen MA, Harder LD. The global stock of domesticated honey bees is growing slower than agricultural demand for pollination. Curr Biol. 2009;19: 915–918. 10.1016/j.cub.2009.03.071 19427214

[4] Potts SG, Biesmeijer JC, Kremen C, Neumann P, Schweiger O, Kunin WE. Global pollinator declines: trends, impacts and drivers. Trends Ecol Evol. 2010;25: 345–353. 10.1016/j.tree.2010.01.007 20188434

[5] Spleen A, Lengerich E, Rennich K, Caron D, Rose R, Pettis JS, et al A national survey of managed honey bee 2011–12 winter colony losses in the United States: results from the Bee Informed Partnership. J Apic Res. 2013;52: 44–53. 10.3896/IBRA.1.52.2.07

[6] Higes M, Martín-Hernández R, Martínez-Salvador A, Garrido-Bailón E, González-Porto AV, Meana A, et al A preliminary study of the epidemiological factors related to honey bee colony loss in Spain. Environ Microbiol Rep. 2010;2: 243–250. 10.1111/j.1758-2229.2009.00099.x 23766075

[7] Genersch E, Ohe von der W, Kaatz H, Schroeder A, Otten C, Büchler R, et al The German bee monitoring project: a long term study to understand periodically high winter losses of honey bee colonies. Apidologie. 2010;41: 332–352. 10.1051/apido/2010014

[8] Rennich K, Pettis J, vanEngelsdorp D, Bozarth R, Eversole H, Evans J, et al. 2011–2012 National honey bee pests and diseases survey report. USDA; 2012.

[9] Pettis JS, vanEngelsdorp D, Johnson J, Dively G. Pesticide exposure in honey bees results in increased levels of the gut pathogen *Nosema* . Naturwissenschaften. 2012;99: 153–158. 10.1007/s00114-011-0881-1 22246149 PMC3264871

[10] Dainat B, vanEngelsdorp D, Neumann P. Colony collapse disorder in Europe. Environ Microbiol Rep. 2011;4: 123–125. 10.1111/j.1758-2229.2011.00312.x 23757238

[11] Higes M, Martín-Hernández R, Botías C, Bailón EG, González-Porto AV, Barrios L, et al How natural infection by *Nosema ceranae* causes honeybee colony collapse. Environ Microbiol. 2008;10: 2659–2669. 10.1111/j.1462-2920.2008.01687.x 18647336

[12] Huang WF, Jiang JH, Chen YW, Wang CH. A *Nosema ceranae* isolate from the honeybee *Apis mellifera* . Apidologie. 2007;38: 30–37. 10.1051/apido:2006054

[13] Chaimanee V, Pettis JS, Chen YP, Evans JD, Khongphinitbunjong K, Chantawannakul P. Susceptibility of four different honey bee species to *Nosema ceranae* . Vet Parasitol. 2012;193: 260–265. 10.1016/j.vetpar.2012.12.004 23290277

[14] Huang W-F, Solter LF, Yau PM, Imai BS. *Nosema ceranae* escapes fumagillin control in honey bees. PLoS Pathog. 2013;9: e1003185 10.1371/journal.ppat.1003185 23505365 PMC3591333

[15] Gisder S, Hedtke K, Möckel N, Frielitz MC, Linde A, Genersch E. Five-year cohort study of *Nosema* spp. in Germany: does climate shape virulence and assertiveness of *Nosema ceranae*? Appl Environ Microbiol. 2010;76: 3032–3038. 10.1128/AEM.03097-09 20228103 PMC2863439

[16] Botías C, Martín-Hernández R, Bailón EG, González-Porto AV, Martínez-Salvador A, De la Rúa P, et al The growing prevalence of *Nosema ceranae* in honey bees in Spain, an emerging problem for the last decade. Res Vet Sci. 2012;93: 150–155. 10.1016/j.rvsc.2011.08.002 21906767

[17] Higes M, Meana A, Bartolomé C, Botías C, Martín-Hernández R. *Nosema ceranae* (Microsporidia), a controversial 21^st^ century honey bee pathogen. Environ Microbiol Rep. 2013;5: 17–29. 10.1111/1758-2229.12024 23757127

[18] Losey JE, Vaughan M. The economic value of ecological services provided by insects. BioScience. 2006;56: 311–323.

[19] Dussaubat C, Brunet JL, Higes M, Colbourne JK, Lopez J, Choi JH, et al Gut pathology and responses to the microsporidium *Nosema ceranae* in the honey bee *Apis mellifera* . PLoS One. 2012;7: e37017 10.1371/journal.pone.0037017.t001 22623972 PMC3356400

[20] Martín-Hernández R, Botías C, Barrios L, Martínez-Salvador A, Meana A, Mayack C, et al Comparison of the energetic stress associated with experimental *Nosema ceranae* and *Nosema apis* infection of honeybees (*Apis mellifera*). Parasitol Res. 2011;109: 605–612. 10.1007/s00436-011-2292-9 21360094

[21] Paxton RJ, Klee J, Korpela S, Fries I. *Nosema ceranae* has infected *Apis mellifera* in Europe since at least 1998 and may be more virulent than *Nosema apis* . Apidologie. 2007;38: 558–565. 10.1051/apido:2007037

[22] Suwannapong G, Maksong S, Seanbualuang P, Benbow ME. Experimental infection of red dwarf honeybee, *Apis florea*, with *Nosema ceranae* . J Asia Pac Entomol. 2010;13: 361–364. 10.1016/j.aspen.2010.07.003

[23] Kralj J, Fuchs S. *Nosema* sp. influences flight behavior of infected honey bee (*Apis mellifera*) foragers. Apidologie. 2009;41: 21–28. 10.1051/apido/2009046

[24] Pettis JS, Lichtenberg EM, Andree M, Stitzinger J, Rose R, vanEngelsdorp D. Crop pollination exposes honey bees to pesticides which alters their susceptibility to the gut pathogen *Nosema ceranae* . PLoS One. 2013;8: e70182 10.1371/journal.pone.0070182.s002 23894612 PMC3722151

[25] Aufauvre J, Biron DG, Vidau C, Fontbonne R, Roudel M, Diogon M, et al Parasite-insecticide interactions: a case study of *Nosema ceranae* and fipronil synergy on honeybee. Sci Rep. 2012;2: 1–7. 10.1038/srep00326 22442753 PMC3310228

[26] Vidau C, Diogon M, Aufauvre J, Fontbonne R, Viguès B, Brunet JL, et al Exposure to sublethal doses of fipronil and thiacloprid highly increases mortality of honeybees previously infected by *Nosema ceranae* . PLoS One. 2011;6: e21550 10.1371/journal.pone.0021550.g004 21738706 PMC3125288

[27] Cameron SA, Mardulyn P. Multiple molecular data sets suggest independent origins of highly eusocial behavior in bees (Hymenoptera: Apinae). Syst Biol. 2001;50: 194–214. 10.1080/10635150120230 12116928

[28] Schmid-Hempel P, Loosli R. A contribution to the knowledge of *Nosema* infections in bumble bees, Bombus spp. Apidologie. 1998;29: 525–536.

[29] Undeen AH, Maddox JV. The infection of nonmosquito hosts by injection with spores of the microsporidan *Nosema algerae* . J Invertebr Pathol. 1973;22: 258–265. 10.1016/0022-2011(73)90143-2 4206297

[30] Blaser M, Schmid-Hempel P. Determinants of virulence for the parasite *Nosema whitei* in its host *Tribolium castaneum* . J Invertebr Pathol. 2005;89: 251–257. 10.1016/j.jip.2005.04.004 15963529

[31] Traver BE, Fell RD. Low natural levels of *Nosema ceranae* in *Apis mellifera* queens. J Invertebr Pathol. 2012;110: 408–410. 10.1016/j.jip.2012.04.001 22546521

[32] Traver BE, Fell RD. *Nosema ceranae* in drone honey bees (*Apis mellifera*). J Invertebr Pathol. 2011;107: 234–236. 10.1016/j.jip.2011.05.016 21621543

[33] Huang WF, Solter LF. Comparative development and tissue tropism of *Nosema apis* and *Nosema ceranae* . J Invertebr Pathol. 2013;113: 35–41. 10.1016/j.jip.2013.01.001 23321524

[34] Higes M, Martín-Hernández R, Garrido-Bailón E, García-Palencia P, Meana A. Detection of infective *Nosema ceranae* (Microsporidia) spores in corbicular pollen of forager honeybees. J Invertebr Pathol. 2008;97: 76–78. 10.1016/j.jip.2007.06.002 17651750

[35] Huang ZY, Otis GW. Inspection and feeding of larvae by worker honey bees (Hymenoptera: Apidae): Effect of starvation and food quantity. J Insect Behav. 1991;4: 305–317. 10.1007/BF01048280

[36] Smith ML. The honey bee parasite *Nosema ceranae*: transmissible via food exchange? PLoS One. 2012;7: e43319 10.1371/journal.pone.0043319 22916241 PMC3420906

[37] Smart MD, Sheppard WS. *Nosema ceranae* in age cohorts of the western honey bee (*Apis mellifera*). J Invertebr Pathol. 2011;109: 148–151. 10.1016/j.jip.2011.09.009 22001631

[38] Spivak MS, Reuter GS. Resistance to American foulbrood disease by honey bee colonies *Apis mellifera* bred for hygienic behavior. Apidologie. 2001;32: 555–565.

[39] Swanson JAI, Torto B, Kells SA, Mesce KA, Tumlinson JH, Spivak M. Odorants that induce hygienic behavior in honeybees: Identification of volatile compounds in chalkbrood-infected honeybee larvae. J Chem Ecol. 2009;35: 1108–1116. 10.1007/s10886-009-9683-8 19816752

[40] Rueppell O, Hayworth MK, Ross NP. Altruistic self-removal of health-compromised honey bee workers from their hive. J Evol Biol. 2010;23: 1538–1546. 10.1111/j.1420-9101.2010.02022.x 20500363

[41] Goblirsch M, Huang ZY, Spivak MS. Physiological and behavioral changes in honey bees (*Apis mellifera*) induced by *Nosema ceranae* infection. PLoS One. 2013;8: e58165 10.1371/journal.pone.0058165.t002 23483987 PMC3590174

[42] Maiolino P, Iafigliola L, Rinaldi L, De Leva G, Restucci B, Martano M. Histopathological findings of the midgut in European honey bee (*Apis mellifera* L.) naturally infected by *Nosema* spp. Vet Med Anim Sci. 2014;2: 1–3. 10.7243/2054-3425-2-4

[43] Higes M, García-Palencia P, Martín-Hernández R, Meana A. Experimental infection of *Apis mellifera* honeybees with *Nosema ceranae* (Microsporidia). J Invertebr Pathol. 2007;94: 211–217. 10.1016/j.jip.2006.11.001 17217954

[44] Suwannapong G, Yemor T, Boonpakdee C, Benbow ME. *Nosema ceranae*, a new parasite in Thai honeybees. J Invertebr Pathol. 2011;102: 236–241. 10.1016/j.jip.2010.10.003 20965196

[45] Fontbonne R, Garnery L, Vidau C, Aufauvre J, Texier C, Tchamitchian S, et al Comparative susceptibility of three Western honeybee taxa to the microsporidian parasite *Nosema ceranae* . Infect Genet Evol. 2013;17: 188–194. 10.1016/j.meegid.2013.04.016 23619100

[46] Huang Z. A standardized procedure for the *in vitro* rearing of honey bee larvae. California Department of Pesticide Regulation Sacramento (CA); 2009.

[47] Cantwell GE. Standard methods for counting *Nosema* spores. American Bee Journal. 1970;110: 222–223.

[48] Hakim RS, Baldwin K, Smagghe G. Regulation of midgut growth, development, and metamorphosis. Annu Rev Entomol. 2010;55: 593–608. 10.1146/annurev-ento-112408-085450 19775239

[49] Cruz-Landim CD, Melo Cavalcante V. Ultrastructural and cytochemical aspects of metamorphosis in the midgut of *Apis mellifera* L. (Hymenoptera: Apidae: Apinae). Zoolog Sci. 2003;20: 1099–1107. 10.2108/zsj.20.1099 14578570

[50] Mayack C, Naug D. Energetic stress in the honeybee *Apis mellifera* from *Nosema ceranae* infection. J Invertebr Pathol 2009;100: 185–188. 10.1016/j.jip.2008.12.001 19135448

[51] Kaftanoglu O, Linksvayer TA, Page RE. Rearing honey bees, *Apis mellifera*, *in vitro* I: effects of sugar concentrations on survival and development. J Insect Sci. 2011;11: 96 10.1673/031.011.9601 22208776 PMC3391908

[52] Kellen WR, Lindegren JE. Biology of *Nosema plodiae* sp. n., a microsporidian pathogen of the Indian-meal moth, *Plodia interpunctella* (Hübner), (Lepidoptera: Phycitidae). J Invertebr Pathol. 1968;11: 104–111. 10.1016/0022-2011(68)90059-1 5654768

[53] Solter LF, Onstad DW, Maddox JV. Timing of disease-influenced processes in the life cycle of *Ostrinia nubilalis* infected with *Nosema pyrausta* . J Invertebr Pathol. 1990;55: 337–341. 10.1016/0022-2011(90)90076-I

[54] Fujiwara T. Infectivity and pathogenicity of *Nosema bombycis* to larvae of the silkworm. J Seric Sci Jpn. 1979; 48: 376–380.

[55] Fisher FM Jr, Sanborn RC. Observations on the susceptibility of some insects to *Nosema* (Microsporidia: Sporozoa). J Parasitol. 1962;48: 926–932. 13945275

[56] van der Steen JJM. Infection and transmission of *Nosema bombi* in *Bombus terrestris* colonies and its effect on hibernation, mating and colony founding. Apidologie. 2008;39: 273–282. 10.1051/apido:2008006

[57] Otti O, Schmid-Hempel P. A field experiment on the effect of *Nosema bombi* in colonies of the bumblebee *Bombus terrestris* . Ecol Entomol. 2008;33: 577–582. 10.1111/j.1365-2311.2008.00998.x

[58] Rutrecht ST, Brown MJF. Within colony dynamics of *Nosema bombi* infections: disease establishment, epidemiology and potential vertical transmission. Apidologie. 2008;39: 504–514. 10.1051/apido:2008031

[59] Bailey L. The infection of the ventriculus of the adult honeybee by *Nosema apis* (Zander). Parasitology. 1955;45: 86–94. 10.1017/S0031182000027451 14370833

[60] Meana AM, Martín-Hernández R, Higes M. The reliability of spore counts to diagnose *Nosema ceranae* infections in honey bees. J Apic Res. 2010;49: 212–214. 10.3896/IBRA.1.49.2.12

[61] Hassanein MH. The influence of *Nosema apis* on the larval honeybee. Ann Appl Biol. 1951;38: 844–846.

[62] Fukuda H, Sakagami SF. Worker brood survival in honeybees. Res Popul Ecol. 1968;10: 31–39.

[63] Di Pasquale G, Salignon M, Le Conte Y, Belzunces LP, Decourtye A, Kretzschmar A, et al Influence of pollen nutrition on honey bee health: do pollen quality and diversity matter? PLoS One. 2013;8: e72016 10.1371/journal.pone.0072016.s003 23940803 PMC3733843

[64] Zhu W, Schmehl DR, Mullin CA, Frazier JL. Four common pesticides, their mixtures and a formulation solvent in the hive environment have high oral toxicity to honey bee larvae. PLoS One. 2014;9 10.1371/journal.pone.0077547 PMC3885384 24416121

[65] Cornman RS, Tarpy DR, Chen YP, Jeffreys L, Lopez D, et al Pathogen webs in collapsing honey bee colonies. PLoS One. 2012;7: e43562 10.1371/journal.pone.0043562 22927991 PMC3424165

[66] vanEngelsdorp D, Speybroeck N, Evans JD, Nguyen BK, Mullin C, Frazier M, et al Weighing risk factors associated with bee colony collapse disorder by classification and regression tree analysis. J Econ Entomol. 2010;103: 1517–1523. 10.1603/EC09429 21061948

[67] Chaimanee V, Chantawannakul P, Chen YP, Evans JD, Pettis JS. Differential expression of immune genes of adult honey bee (*Apis mellifera*) after inoculated by *Nosema ceranae* . J Insect Physiol. 2012;58: 1090–1095. 10.1016/j.jinsphys.2012.04.016 22609362

[68] Forsgren E, Fries I. Comparative virulence of *Nosema ceranae* and *Nosema apis* in individual European honey bees. Vet Parasitol. 2010;170: 212–217. 10.1016/j.vetpar.2010.02.010 20299152

[69] Huang Q, Kryger P, Le Conte Y, Moritz RFA. Survival and immune response of drones of a *Nosemosis* tolerant honey bee strain towards *N*. *ceranae* infections. J Invertebr Pathol. 2012;109: 297–302. 10.1016/j.jip.2012.01.004 22285444

[70] Webster TC, Pomper KW, Hunt G, Thacker EM, Jones SC. *Nosema apis* infection in worker and queen *Apis mellifera* . Apidologie. 2004;35: 49–54. 10.1051/apido:2003063

[71] Gisder S, Genersch E. Molecular differentiation of *Nosema apis* and *Nosema ceranae* based on species-specific sequence differences in a protein coding gene. J Invertebr Pathol. 2013;113: 1–6. 10.1016/j.jip.2013.01.004 23352902

[72] Winston ML. The biology of the honey bee. Cambridge, Massachusetts: Harvard University Press; 1987

[73] Crailsheim K, Brodschneider R, Aupinel P, Behrens D, Genersch E, Vollmann J, et al Standard methods for artificial rearing of *Apis mellifera* larvae. J Apic Res. 2013;52: 1–15. 10.3896/IBRA.1.52.1.05

[74] Huang Q, Kryger P, Le Conte Y, Lattorff HMG, Kraus FB, Moritz RFA. Four quantitative trait loci associated with low *Nosema ceranae* (Microsporidia) spore load in the honeybee *Apis mellifera* . Apidologie. 2014;45: 248–256. 10.1007/s13592-013-0243-4

[75] Fries I, Chauzat M-PP, Chen YP, Doublet V, Genersch E, Gisder S, et al Standard methods for *Nosema* research. J Apic Res. 2013;52: 1–28. 10.3896/IBRA.1.52.1.14

[76] Williams GR, Alaux C, Costa C, Csáki T, Doublet V, Eisenhardt D, et al Standard methods for maintaining adult *Apis mellifera* in cages under *in vitro* laboratory conditions. J Apic Res. 2013;52: 1–34. 10.3896/IBRA.1.52.1.04

[77] Dadant. The hive and the honeybee. Hamilton, Illinois: Dadant & Sons; 1975.

